# Longitudinal analysis of insulin resistance and sarcopenic obesity in Chinese middle-aged and older adults: evidence from CHARLS

**DOI:** 10.3389/fpubh.2024.1472456

**Published:** 2024-11-18

**Authors:** Chunyan Xu, Ling He, Yansong Tu, Canhui Guo, Hurong Lai, Caifeng Liao, Chuyang Lin, Huaijun Tu

**Affiliations:** ^1^The Second Affiliated Hospital, Jiangxi Medical College, Nanchang University, Nanchang, China; ^2^The Department of Geriatrics, The Second Affiliated Hospital of Nanchang University, Nanchang, China; ^3^Faculty of Science, The University of Melbourne, Parkville, VIC, Australia

**Keywords:** sarcopenic obesity, China Health and Retirement Longitudinal Study, surrogate insulin resistance indices, TyG-waist-to-height ratio, Chinese middle-aged and older adults

## Abstract

**Objective:**

The correlation between surrogate insulin resistance (IR) indices and sarcopenic obesity (SO) remains uncertain. This study aimed to assess the association between six IR surrogates—triglyceride-glucose (TyG), TyG-waist circumference (TyG-WC), TyG-waist-to-height ratio (TyG-WHtR), triglyceride-to-high-density lipoprotein-cholesterol ratio (TG/HDL), metabolic score for insulin resistance (METS-IR), and Chinese visceral adiposity index (CVAI)—and SO risk in a middle-aged and older population in China.

**Methods:**

The study employed longitudinal data obtained from the China Health and Retirement Longitudinal Study (CHARLS) between 2011 and 2015, involving 6,395 participants. We used multivariate logistic regression models to examine the link between six surrogates and SO. Nonlinear relationships were evaluated using restricted cubic spline analysis, and subgroup analyses were conducted for validation. Receiver operating characteristic (ROC) curves were used to assess predictive capabilities.

**Results:**

Over the course of a 4-year follow-up period, 319 participants (5.0%) developed SO. In the fully adjusted model, all six surrogates were significantly associated with SO. The adjusted odds ratios (ORs) with a 95% confidence interval (95% CI) per standard deviation increase were 1.21 (1.08–1.36) for TyG, 1.56 (1.39–1.75) for TyG-WC, 2.04 (1.81–2.31) for TyG-WHtR, 1.11 (1.01–1.21) for TG/HDL, 1.67 (1.50–1.87) for METS-IR, and 1.74 (1.55–1.97) for CVAI. Notably, TyG-WC, TyG-WHtR, TG/HDL, METS-IR, and CVAI exhibited nonlinear correlations with SO. Conversely, TG/HDL did not exhibit a significant association during subgroup analysis. Furthermore, TyG-WHtR had a significantly larger area under the receiver operating characteristic curve than other indices.

**Conclusion:**

The results indicated that TyG, TyG-WC, TyG-WHtR, METS-IR, and CVAI were significantly and positively associated with SO incidence. Meanwhile, TyG-WC, TyG-WHtR, METS-IR, and CVAI showed nonlinear relationships with SO. Specifically, TyG-WHtR may be the most appropriate indicator for predicting SO among middle-aged and older Chinese adults.

## 1 Introduction

As its name suggests, the clinical condition known as sarcopenic obesity (SO) arises from the concurrent presence of sarcopenia and obesity. As individuals age, there is often an increase in adipose tissue and a reduction in muscle mass, which can accelerate the progression of SO. SO has a greater negative impact on human health than either obesity or sarcopenia in isolation. Previous studies have shown that SO is associated with an increased risk of developing various metabolic diseases, including diabetes (1), hypertension (2), and metabolic syndrome (3). Furthermore, individuals with SO are prone to develop frailty, disability, and even mortality (4). The prevalence of SO is estimated to be between 2.75 and 20% (5). The current reported prevalence of SO varies considerably, which may be related to the different diagnostic criteria used. Due to the increasing trend of population aging, the prevalence of SO is anticipated to increase. Therefore, early detection and prevention of SO may prove to be an effective strategy for reducing the burden of disease and alleviating global public health pressures.

Insulin resistance (IR) is defined as an impaired ability of insulin to assist in the absorption and utilization of glucose, resulting in compensatory hyperinsulinemia as the body attempts to maintain glucose homeostasis. Individuals with insulin resistance often present with metabolic disturbances, including hyperglycemia and hyperlipidemia. These conditions accelerate muscle breakdown and exacerbate fat accumulation, thereby promoting the development of SO (6, 7). Various methodologies have been established to evaluate IR, with the Homeostasis Model Assessment of Insulin Resistance (HOMA-IR) emerging as a prevalent technique. Prior research has provided empirical evidence corroborating that increased HOMA-IR values are significantly and independently associated with an increased risk of SO (8–11), which highlights the potential of HOMA-IR as a strong prognostic biomarker. Nevertheless, the requirement for fasting insulin levels limits the applicability of HOMA-IR in clinical situations. In recent years, alternative indices of IR that do not require insulin measurement have received increasing attention and have been demonstrated to be reliable tools for the assessment of IR. These include the triglyceride-glucose (TyG) index (12), the ratio of triglyceride-to-high-density lipoprotein-cholesterol (TG/HDL) (13), the TyG index adjusted for waist circumference (TyG-WC) (14), the TyG index adjusted for waist-to-height ratio (TyG-WHtR) (14), the metabolic score for IR (METS-IR) (15), and the Chinese visceral adiposity index (CVAI) (16).

Recent research suggests that the TyG index may serve as a valuable predictor of SO (17). Nevertheless, there is a lack of research examining the correlation between IR surrogate indicators and SO, and it remains unclear whether a nonlinear relationship exists between these indicators and SO. To address this gap, this study used the national longitudinal survey of middle-aged and older adults database to investigate the correlations between six IR surrogate indicators and SO risk and to evaluate and compare the diagnostic value of different IR surrogate indicators for new-onset SO.

## 2 Materials and methods

### 2.1 Study design and population

The China Health and Retirement Longitudinal Study (CHARLS) is a nationally representative longitudinal study that focuses on the aging process among Chinese community members aged 45 years and older. The results of the study are available to the public for free.^1^ The baseline survey of the CHARLS was initiated in 2011 (Wave 1), and a total of 17,708 participants were enrolled across 150 counties or districts and 450 villages encompassing 28 provinces throughout China. Following the baseline survey, biennial follow-up surveys were conducted, with Waves 2 in 2013, 3 in 2015, and 4 in 2018. The database contains a comprehensive array of information, including demographic characteristics, health status, physical measurements, family structures, financial status, and other pertinent information. Blood samples were collected during Waves 1 and 3 in order to supplement the research. The methodologies regarding sampling, questionnaire construction, and clinical assessments have been documented in previous reports (18). Prior to their participation, all individuals provided written informed consent, and the study received ethical approval from the Institutional Review Board of Peking University (IRB00001052-11015).

The initial sample of this investigation consisted of 17,708 individuals from the baseline survey (Wave 1). To maintain the integrity and relevance of the study cohort, a set of exclusion criteria were meticulously applied. Individuals were deemed ineligible for the study if they lacked baseline measurements for any six IR indices (*n* = 7,958), if their age data was incomplete, or if they were under 45 years of age (*n* = 339). Moreover, participants with a confirmed diagnosis of SO at baseline or with incomplete information regarding SO were excluded (*n* = 508). Further exclusions were made for those lacking SO data in the subsequent Waves 2 and 3 (*n* = 1,988), for those with atypical Body mass index (BMI) and waist measurements (*n* = 178), and for those without comprehensive demographic, health behavior, and chronic disease information (*n* = 342). Following the application of these exclusionary criteria, the final dataset used for analysis consisted of 6,395 participants (Supplementary Figure S1).

### 2.2 Evaluation of six surrogate insulin resistance indices

Venous blood samples were collected from each participant following an overnight fast, conducted by professional medical personnel from the Chinese Center for Disease Control and Prevention. Thereafter, the samples were then subjected to centrifugation before being transported to the laboratory at Capital Medical University for further analysis. The blood lipids and glucose levels were determined through the use of enzymatic colorimetric assays. Anthropometric measurements, including body weight and height, were recorded with precision using an Omron HN-286 scale and a Seca 213 stadiometer, respectively, with accuracy to the nearest 0.1 unit. Body mass index (BMI) was calculated by dividing weight in kilograms by height in meters squared. At the conclusion of a gentle exhalation, waist circumference was measured at the umbilical level. The surrogate IR indices were calculated using specific formulas based on the established methodologies of previous studies (12–16), and these six formulas are specified as follows [Equations 1-6]:


(1) $$TyG=Ln\;\left[\;\left(TG\;\left(\mathrm{mg}/\mathrm{dL}\right)\times \mathrm{glucose}\;\left(\mathrm{mg}/\mathrm{dL}\right)/2\right)\;\right]$$

(2) $$\begin{array}{l} TyG-WC\\ =Ln\;\left[\;\left(TG\;\left(\mathrm{mg}/\mathrm{dL}\right)\times \mathrm{glucose}\;\left(\mathrm{mg}/\mathrm{dL}\right)/2\right)\;\right]\times WC\;\left(\mathrm{cm}\right) \end{array}$$

(3) $$\begin{array}{l} TyG-\mathrm{WHtR}\\ =Ln\;\left[\;\left(TG\;\left(\mathrm{mg}/\mathrm{dL}\right)\times \mathrm{glucose}\;\left(\mathrm{mg}/\mathrm{dL}\right)/2\right)\;\right]\\ \times \left[\;WC\;\left(\mathrm{cm}\right)/\mathrm{Height}\;\left(m\right)\;\right] \end{array}$$

(4) $$TG/HDL=TG\;\left(\mathrm{mg}/\mathrm{dL}\right)/HDL-c\;\left(\mathrm{mg}/\mathrm{dL}\right)$$

(5) $$\begin{array}{l} \mathrm{METS}-IR=Ln\;\left(2\times \mathrm{glucose}\;\left(\mathrm{mg}/\mathrm{dL}\right)+TG\;\left(\mathrm{mg}/\mathrm{dL}\right)\right)\\ \;\times BMI\;\left(\mathrm{kg}/m^{2}\right)/Ln\;\left(HDL-c\;\left(\mathrm{mg}/\mathrm{dL}\right)\right) \end{array}$$

Males:


$$\begin{array}{l} \mathrm{CVAI}=–267.93+0.68\times \mathrm{age}\;\left(\mathrm{years}\right)+0.03\times BMI\;\left(\mathrm{kg}/m^{2}\right)+\\ \;4.00\times WC\;\left(\mathrm{cm}\right)+22.00\times \log_{10}TG\;\left(\mathrm{mmol}/L\right)–\\ \;16.32\times HDL-c\;\left(\mathrm{mmol}/L\right) \end{array}$$

Females:


(6) $$\begin{array}{l} \mathrm{CVAI}=–187.32+1.71\times \mathrm{age}\;\left(\mathrm{years}\right)+4.23\times BMI\;\left(\mathrm{kg}/m^{2}\right)\\ \;+\;1.12\times WC\;\left(\mathrm{cm}\right)+39.76\times \log_{10}TG\;\left(\mathrm{mmol}/L\right)–\\ \;11.66\times HDL-c\;\left(\mathrm{mmol}/L\right) \end{array}$$

### 2.3 Assessment of sarcopenic obesity

Sarcopenic obesity (SO) is defined as the coexistence of both sarcopenia and obesity (19, 20). In this study, obesity was categorized according to the modified Asia-Pacific criteria, with a BMI of 25 kg/m^2^ or higher being an indicator (21). It has been recognized that the BMI alone has limitations, particularly in individuals of varying heights, where taller individuals may not be obese despite having a higher BMI, whereas shorter individuals may appear overweight. To address this issue, we combined BMI with appendicular skeletal muscle mass (ASM) to provide a more comprehensive assessment. The diagnosis of sarcopenia is based on the criteria developed by the Foundation for the National Institutes of Health (FNIH) (22). To quantify ASM, we employed an established anthropometric formula specific to the Chinese population, which has shown high consistency with dual-energy X-ray absorptiometry (DXA) measurements (23, 24). The calculation formula for ASM is as follows (23):


$$\begin{array}{l} ASM=0.193\times \mathrm{weight}\;\left(\mathrm{kg}\right)+0.107\times \mathrm{height}\;\left(\mathrm{cm}\right)\\ \;-4.157\times \mathrm{sex}-0.037\times \mathrm{age}\;\left(\mathrm{years}\right)-2.631\text{.} \end{array}$$

where sex code is 1 for males and 2 for females. This threshold for “sarcopenia,” i.e., adjusted skeletal muscle mass based on body mass index (BMI) (ASM/BMI), is less than 0.512 for females and 0.789 for males (22). This combined approach mitigates the potential misclassification due to height-related variability.

### 2.4 Covariates

The selection of covariates in our study was informed by both empirical evidence from preceding scholarly studies and the informed judgment of clinical professionals (25–28). The covariates were classified into the following categories: (1) categorical variables: gender, marriage, residence, educational attainment, smoking habits, alcohol consumption, and chronic diseases, including hypertension, diabetes mellitus, dyslipidemia, cancer, pulmonary disease, heart disease, stroke, liver disease, renal disease, and depression. (2) Continuous variables: age, duration of sleep, BMI, waist circumference (WC), C-reactive protein (CRP), serum high-density lipoprotein cholesterol (HDL-c), serum low-density lipoprotein cholesterol (LDL-c), triglycerides (TG), total serum cholesterol (TC), and serum creatinine.

### 2.5 Statistical analysis

The characteristics of the participants at the baseline stage were subjected to analysis with the aim of identifying those who were newly diagnosed with SO. Continuous variables with a normal distribution were expressed as mean ± standard deviation (SD), while variables with an irregular distribution were depicted as median and interquartile range (IQR). Categorical variables were presented as counts, while percentages (%) were presented for categorical variables. For continuous variables, comparative analyses across groups were performed using Student’s *t*-tests or Mann–Whitney U tests and chi-square tests for categorical data.

Prior to developing logistic regression models, we computed the generalized variance inflation factor to investigate the possibility of multicollinearity between six IR surrogates and other factors. Due to the significant multicollinearity among the six surrogate IR indices, as detailed in Supplementary Table S1, BMI, WC, TC, TG, LDL, and HDL were excluded from the final logistic regression models. To investigate the correlation between the baseline surrogate IR indices and the likelihood of incident SO, univariate and multivariate logistic regression analyses were employed. Three additional models constructed were as follows: Model 1 (without adjustment), Model 2 (with adjustments for demographics, including age, gender, marital status, living situation, and educational attainment), and Model 3 (with adjustments for additional lifestyle factors and health indicators such as smoking, drinking, sleep duration, chronic diseases, CRP, and creatinine based on Model 2). Moreover, an investigation was conducted to examine the dose–response correlation between the per-SD increases in the surrogate IR indices and SO incidence. Nonlinear relationships were examined using adjusted cubic splines, and inflection points were identified using a recursive algorithm. Afterward, two-piecewise logistic regression models were then formulated around these inflection points. The most suitable models were identified using log-likelihood ratio tests to elucidate the link between IR surrogates and SO.

Subgroup and interaction analyses were conducted to investigate the association between IR surrogates and SO risk with stratification by gender and age. The receiver operating characteristic (ROC) curve was employed to assess the prediction accuracy of six IR surrogates for SO, and differences in the area under the curve (AUC) values were compared using z-tests.

All statistical analyses were executed using SPSS version 25.0 (IBM SPSS, Armonk, NY, United States) and R version 4.2.3 (R Development Core Team, Vienna, Austria). A two-tailed test with a significance level of *p* < 0.05 was used to determine whether the observed differences in the statistics were statistically significant.

## 3 Results

### 3.1 Baseline characteristics

A total of 6,395 individuals, initially unaffected by SO, were continuously monitored till 2015. The baseline characteristics of the cohort are detailed in Table 1, which are differentiated by the frequency of SO throughout the follow-up period. During the four-year observation period, 319 individuals (5.0%) developed new-onset SO. Individuals with SO were more likely to be older, male, urban dwellers, and to consume alcohol, as well as to have increased levels of BMI, WC, CRP, TC, TG, and creatinine, along with decreased HDL levels. This group also experienced a higher prevalence of comorbidities such as hypertension, cardiovascular disease, and stroke. Furthermore, increased values of all six IR surrogates were observed in these participants.

**Table 1:** Baseline characteristics of study participants according to sarcopenic obesity.

Variable	Total (*n* = 6,395)	Non-SO (*n* = 6,076)	SO (*n* = 319)	*p* Value
Age (years)	58 [52–64]	58 [52–64]	61 [55–68]	<0.001
Gender (%)				0.007
Male	2,942 (46.0)	2,772 (45.6)	170 (53.3)	
Female	3,453 (54.0)	3,304 (54.4)	149 (46.7)	
Marriage (%)				0.073
Yes	5,436 (85.0)	5,176 (85.2)	260 (81.5)	
Others	959 (15.0)	900 (14.8)	59 (18.5)	
Residence (%)				0.012
Urban	2,057 (32.2)	1,934 (31.8)	123 (38.6)	
Rural	4,338 (67.8)	4,142 (68.2)	196 (61.4)	
Education (%)				0.054
Primary school or below	4,523 (70.7)	4,279 (70.4)	244 (76.5)	
Middle school	1,275 (19.9)	1,221 (20.1)	54 (16.9)	
High school or above	597 (9.3)	576 (9.5)	21 (6.6)	
Smoking (%)	2,478 (38.7)	2,341 (38.5)	137 (42.9)	0.114
Drinking (%)	2,463 (38.5)	2,323 (38.2)	140 (43.9)	0.043
Sleep duration (hours)	6.50 [5.00–8.00]	7.00 [5.00–8.00]	6.00 [5.00–8.00]	0.137
Chronic diseases (%)				
Hypertension	1,611 (25.2)	1,495 (24.6)	116 (36.4)	<0.001
Diabetes Mellitus	372 (5.8)	357 (5.9)	15 (4.7)	0.383
Dyslipidemia	610 (9.5)	571 (9.4)	39 (12.2)	0.094
Cancer	49 (0.8)	44 (0.7)	5 (1.6)	0.176
Pulmonary disease	604 (9.4)	577 (9.5)	27 (8.5)	0.539
Heart disease	738 (11.5)	684 (11.3)	54 (16.9)	0.002
Stroke	137 (2.1)	124 (2.0)	13 (4.1)	0.014
Liver disease	214 (3.3)	200 (3.3)	14 (4.4)	0.288
Kidney disease	369 (5.8)	346 (5.7)	23 (7.2)	0.258
Depression	1,830 (28.6)	1,731 (28.5)	99 (31.0)	0.327
BMI (kg/m^2^)	22.95 [20.77–25.36]	22.80 [20.66–25.27]	24.64 [23.49–26.47]	<0.001
WC (cm)	84.20 [77.80–91.40]	84.00 [77.40–91.20]	89.30 [84.00–95.00]	<0.001
CRP (mg/dL)	0.98 [0.54–2.07]	0.97 [0.53–2.00]	1.31 [0.71–2.61]	<0.001
HDL (mg/dL)	49.48 [40.79–59.92]	49.87 [40.98–60.31]	47.17 [37.69–55.86]	<0.001
LDL (mg/dL)	114.05 [93.17–136.47]	114.05 [92.78–136.47]	115.21 [96.84–138.02]	0.332
TC (mg/dL)	190.98 [167.40–214.56]	190.59 [167.01–214.56]	191.75 [170.10–217.85]	0.267
TG (mg/dL)	103.54 [74.34–152.22]	103.54 [74.34–150.67]	112.39 [80.98–165.94]	0.002
Creatinine (mg/dL)	0.76 [0.64–0.88]	0.76 [0.64–0.87]	0.78 [0.68–0.91]	<0.001
Surrogate IR indices				
TyG	8.59 [8.22–9.02]	8.58 [8.21–9.02]	8.68 [8.36–9.16]	0.001
TyG-WC	724.66 [650.99–813.15]	720.81 [648.36–810.21]	773.13 [716.55–854.76]	<0.001
TyG-WHtR	459.78 [411.20–514.29]	456.57 [409.24–511.22]	508.06 [462.00–564.70]	<0.001
TG/HDL	2.08 [1.30–3.53]	2.06 [1.29–3.49]	2.35 [1.56–4.11]	<0.001
METS-IR	33.84 [29.45–39.48]	33.58 [29.28–39.30]	37.43 [34.33–42.33]	<0.001
CVAI	90.85 [66.50–119.96]	89.64 [65.45–118.64]	113.70 [91.71–139.88]	<0.001

SO, sarcopenic obesity; BMI, body mass index; WC, waist circumference; CRP, C-reactive protein; HDL, high-density lipoprotein cholesterol; LDL, low-density lipoprotein cholesterol; TC, total serum cholesterol; TG, triglycerides; IR, insulin resistance; TyG, triglyceride-glucose; TyG-WC, triglyceride-glucose-waist circumference; TyG-WHtR, triglyceride-glucose-waist-to-height ratio; TG/HDL, triglyceride-to-high-density-lipoprotein-cholesterol ratio; METS-IR, metabolic score for insulin resistance; CVAI, Chinese visceral adiposity index.

### 3.2 Association of baseline surrogate IR indices with onset risk of SO based on logistic regression

Supplementary Figure S2 shows the correlation between the quartiles of six surrogates and the prevalence of SO. It can be observed that the incidence of SO increases with higher levels of IR surrogates. Table 2 shows the odds ratios (ORs) and corresponding 95% confidence intervals (CIs) for SO across varying quartiles as well as for a per-SD increase in the levels of six surrogate IR indices. Using the adjustment potential confounders as per Model 3, a per-SD increase increment in each index was linked to adjusted ORs of 1.21 (1.08–1.36) for TyG, 1.56 (1.39–1.75) for TyG-WC, 2.04 (1.81–2.31) for TyG-WHtR, 1.11 (1.01–1.21) for TG/HDL, 1.67 (1.50–1.87) for METS-IR, and 1.74 (1.55–1.97) for CVAI. A comprehensive account of the relationship between the indices’ quartiles and SO risk, including the impact of each surrogate IR index, is provided in Table 2 and shown in Supplementary Figure S2.

**Table 2:** Logistic analysis between six surrogate IR indices and SO incidence.

	Model 1	*p* Value	Model 2	*p* Value	Model 3	*p* Value
TyG						
Per-SD increase	1.20 [1.08–1.34]	<0.001	1.23 [1.10–1.37]	<0.001	1.21 [1.08–1.36]	<0.001
Q1	Reference		Reference		Reference	
Q2	1.50 [1.05–2.14]	0.027	1.56 [1.09–2.23]	0.015	1.52 [1.06–2.18]	0.022
Q3	1.86 [1.32–2.62]	<0.001	1.94 [1.37–2.74]	<0.001	1.86 [1.31–2.63]	<0.001
Q4	1.78 [1.26–2.52]	0.001	1.90 [1.34–2.69]	<0.001	1.77 [1.24–2.54]	0.002
TyG-WC						
Per-SD increase	1.50 [1.35–1.67]	<0.001	1.56 [1.40–1.73]	<0.001	1.56 [1.39–1.75]	<0.001
Q1	Reference		Reference		Reference	
Q2	2.08 [1.33–3.23]	0.001	2.19 [1.41–3.42]	<0.001	2.15 [1.38–3.37]	<0.001
Q3	3.90 [2.59–5.87]	<0.001	4.28 [2.83–6.47]	<0.001	4.20 [2.77–6.38]	<0.001
Q4	4.14 [2.76–6.22]	<0.001	4.69 [3.10–7.11]	<0.001	4.54 [2.96–6.96]	<0.001
TyG-WHtR						
Per-SD increase	1.76 [1.59–1.95]	<0.001	1.98 [1.77–2.22]	<0.001	2.04 [1.81–2.31]	<0.001
Q1	Reference		Reference		Reference	
Q2	2.87 [1.71–4.80]	<0.001	3.30 [1.96–5.54]	<0.001	3.32 [1.97–5.58]	<0.001
Q3	4.88 [3.00–7.95]	<0.001	6.09 [3.72–9.99]	<0.001	6.12 [3.72–10.08]	<0.001
Q4	8.19 [5.11–13.13]	<0.001	11.69 [7.17–19.06]	<0.001	12.04 [7.32–19.80]	<0.001
TG/HDL						
Per-SD increase	1.11 [1.02–1.21]	0.014	1.12 [1.03–1.22]	0.008	1.11 [1.01–1.21]	0.025
Q1	Reference		Reference		Reference	
Q2	1.43 [1.01–2.03]	0.044	1.49 [1.05–2.11]	0.027	1.47 [1.03–2.09]	0.033
Q3	1.51 [1.07–2.13]	0.020	1.58 [1.11–2.24]	0.010	1.52 [1.07–2.16]	0.020
Q4	1.86 [1.33–2.60]	<0.001	2.02 [1.44–2.83]	<0.001	1.87 [1.32–2.64]	<0.001
METS-IR						
Per-SD increase	1.51 [1.37–1.66]	<0.001	1.66 [1.50–1.84]	<0.001	1.67 [1.50–1.87]	<0.001
Q1	Reference		Reference		Reference	
Q2	2.74 [1.61–4.66]	<0.001	3.07 [1.80–5.24]	<0.001	3.12 [1.83–5.32]	<0.001
Q3	7.00 [4.29–11.40]	<0.001	9.06 [5.52–14.86]	<0.001	9.21 [5.59–15.17]	<0.001
Q4	7.07 [4.34–11.51]	<0.001	9.94 [6.03–16.38]	<0.001	10.16 [6.10–16.94]	<0.001
CVAI						
Per-SD increase	1.79 [1.61–2.00]	<0.001	1.74 [1.55–1.94]	<0.001	1.74 [1.55–1.97]	<0.001
Q1	Reference		Reference		Reference	
Q2	2.65 [1.61–4.35]	<0.001	2.84 [1.72–4.69]	<0.001	2.86 [1.73–4.73]	<0.001
Q3	4.79 [3.00–6.63]	<0.001	5.14 [3.20–8.25]	<0.001	5.12 [3.18–8.24]	<0.001
Q4	6.88 [4.37–10.85]	<0.001	6.85 [4.31–10.89]	<0.001	6.71 [4.18–10.77]	<0.001

Model 1: Unadjusted model. Model 2: Adjusted for age, sex, marriage, residence, and education. Model 3: further adjusted for smoking, drinking, sleep duration, chronic diseases, CRP, and creatinine based on model 2.

TyG, triglyceride-glucose; TyG-WC, triglyceride-glucose-waist circumference; TyG-WHtR, triglyceride-glucose-waist-to-height ratio; TG/HDL, triglyceride-to-high-density-lipoprotein-cholesterol ratio; METS-IR, metabolic score for insulin resistance; CVAI, Chinese visceral adiposity index.

### 3.3 Nonlinear relationship between baseline surrogate IR indices and onset risk of SO

**Figure 1:**
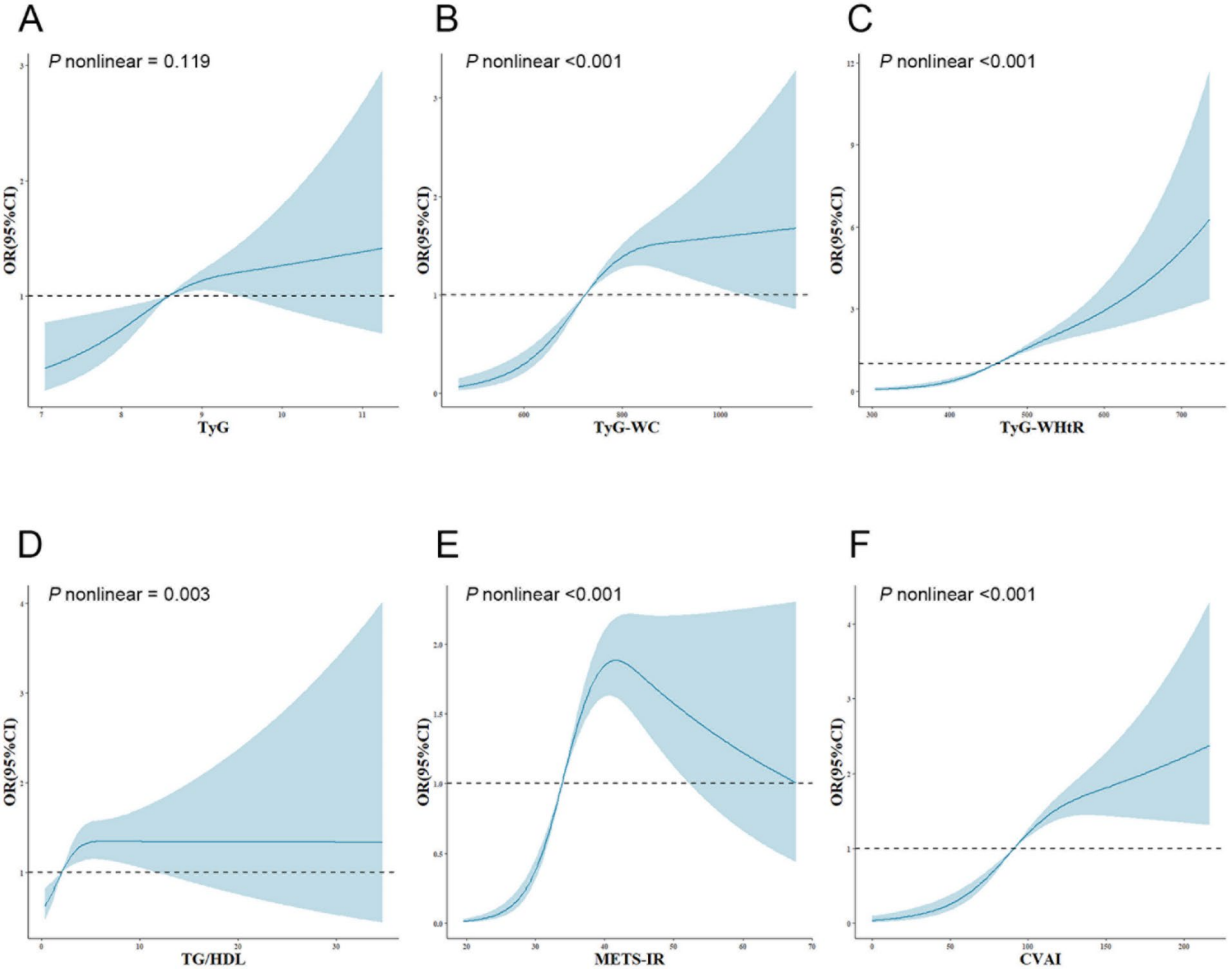
Adjusted cubic spline model of the relationship between six surrogate IR indices and SO risk. All models were adjusted for various factors, including age, sex, marriage, residence, education, smoking, drinking, sleep duration, chronic diseases, CRP, and creatinine. The solid line and blue area represent the estimated adjusted OR and 95% CI, respectively. The horizontal dotted line represents the odds ratio of 1.0. **(A)** TyG index, **(B)** TyG-WC, **(C)** TyG-WHtR, **(D)** TG/HDL, **(E)** METS-IR, **(F)** CVAI. TyG, triglyceride-glucose; TyG-WC, triglyceride-glucose-waist circumference; TyG-WHtR, triglyceride-glucose-waist-to-height ratio; TG/HDL, triglyceride-to-high-density-lipoprotein-cholesterol ratio; METS-IR, metabolic score for insulin resistance; CVAI, Chinese visceral adiposity index; OR, odds ratio; 95%CI, 95% confidence interval.

The dose–response relationship between IR surrogates and SO is illustrated and examined using restricted cubic splines, as shown in Figure 1. After adjusting for numerous confounding variables in Model 3, the TyG index showed a linear correlation with the emergence of SO (P nonlinear = 0.119). Conversely, the TyG-WC, TyG-WHtR, TG/HDL, METS-IR, and CVAI indices revealed notable nonlinear correlations with SO (P nonlinear <0.001; P nonlinear <0.001; P nonlinear = 0.003; P nonlinear <0.001; P nonlinear <0.001).

Subsequently, a threshold effect analysis was conducted to identify the inflection points in the connections between TyG-WC, TyG-WHtR, TG/HDL, METS-IR, and CVAI and the risk of SO. A two-piecewise logistic regression model was employed to assess the ORs and CIs on either side of these demarcations, as detailed in Table 3. The inflection points for TyG-WC, TG/HDL, METS-IR, and CVAI were 780.53, 15.52, 40.95, and 100.14, respectively. When TyG-WC, TG/HDL, METS-IR, and CVAI were below their respective inflection points, a significant positive association with SO risk was observed, with ORs of 1.010 (95% CI: 1.007–1.013, *p* < 0.001), 1.074 (95% CI: 1.029–1.121, *p* = 0.001), 1.191 (95% CI: 1.150–1.233, *p* < 0.001), and 1.036 (95% CI: 1.024–1.048, *p* < 0.001). Following the inflection points, the correlations were not statistically significant, with ORs of 1.002 (95% CI: 0.999–1.004, *p* = 0.165), 0.009 (95% CI: 0.000 to infinity, *p* = 0.994), 0.981 (95% CI: 0.937–1.027, *p* = 0.416), and 1.005 (95% CI: 0.998–1.011, *p* = 0.160). For TyG-WHtR, the OR was 1.017 (95% CI: 1.012–1.023, *p* < 0.001) before and 1.006 (95% CI: 1.003–1.010, *p* < 0.001) after the inflection point of 493.67.

**Table 3:** Threshold effect analysis of surrogate IR indices on SO.

	Adjusted OR (95%CI)	*p* Value
TyG-WC		
Fitting model by standard linear regression	1.004 (1.003–1.005)	**<**0.001
Inflection point	780.53	
<780.53	1.010 (1.007–1.013)	**<**0.001
≥780.53	1.002 (0.999–1.004)	0.165
P for log-likelihood ratio test	**<**0.001	
TyG-WHtR		
Fitting model by standard linear regression	1.010 (1.008–1.011)	**<**0.001
Inflection point	493.67	
<493.67	1.017 (1.012–1.023)	**<**0.001
≥493.67	1.006 (1.003–1.010)	**<**0.001
P for log-likelihood ratio test	**<**0.001	
TG/HDL		
Fitting model by standard linear regression	1.029 (1.004–1.055)	0.025
Inflection point	15.52	
<15.52	1.074 (1.029–1.121)	0.001
≥15.52	0.009 (0.000–inf)	0.994
P for log-likelihood ratio test	0.004	
METS-IR		
Fitting model by standard linear regression	1.069 (1.054–1.084)	**<**0.001
Inflection point	40.95	
<40.95	1.191 (1.150–1.233)	**<**0.001
≥40.95	0.981 (0.937–1.027)	0.416
P for log-likelihood ratio test	**<**0.001	
CVAI		
Fitting model by standard linear regression	1.015 (1.011–1.018)	**<**0.001
Inflection point	100.14	
<100.14	1.036 (1.024–1.048)	**<**0.001
≥100.14	1.005 (0.998–1.011)	0.160
P for log-likelihood ratio test	**<**0.001	

Odds ratios were adjusted for age, sex, marriage, residence, education, smoking, drinking, sleep duration, chronic diseases, CRP, and creatinine.

Inf, infinity; OR, odds ratio; 95%CI, 95% confidence interval; TyG, triglyceride-glucose; TyG-WC, triglyceride-glucose-waist circumference; TyG-WHtR, triglyceride-glucose-waist-to-height ratio; TG/HDL, triglyceride-to-high-density-lipoprotein-cholesterol ratio; METS-IR, metabolic score for insulin resistance; CVAI, Chinese visceral adiposity index.

### 3.4 Subgroup analysis according to gender and age

**Figure 2:**
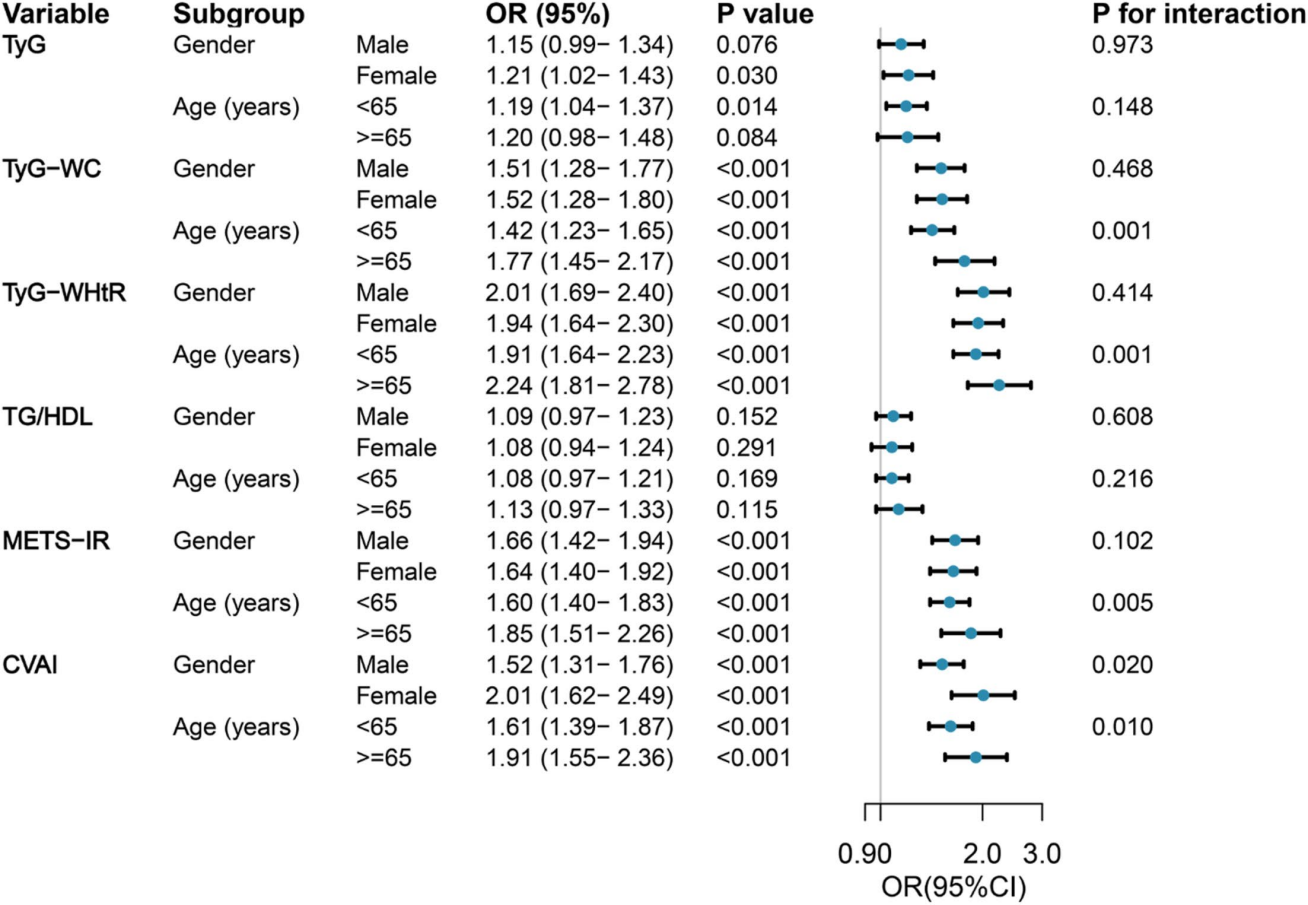
Forest maps of the association between six surrogate IR indices and the risk of incident SO by age and gender. Odds ratios were adjusted for age, sex, marriage, residence, education, smoking, drinking, sleep duration, chronic diseases, CRP, and creatinine. OR, odds ratio; 95%CI, 95% confidence interval; TyG, triglyceride-glucose; TyG-WC, triglyceride-glucose-waist circumference; TyG-WHtR, triglyceride-glucose-waist-to-height ratio; TG/HDL, triglyceride-to-high-density lipoprotein-cholesterol ratio; METS-IR, metabolic score for insulin resistance; CVAI, Chinese visceral adiposity index.

Figure 2 shows the results of the subgroup analysis, stratified by gender and age, regarding the correlation between six IR surrogates and the incidence of SO. In the various subgroups, significant positive correlations were observed between SO risk and TyG-WC, TyG-WHtR, METS-IR, and CVAI, as demonstrated. In contrast, no discernible correlation was observed between TG/HDL and SO risk in any of the subgroups, and no interactions with age or gender were identified. It is noteworthy that the correlation between TyG-WC, TyG-WHtR, METS-IR, CVAI, and SO was more robust in older individuals than in those of middle age. Female participants demonstrated an especially high correlation between CVAI and SO. In the course of this investigation, age was identified as an important interacting factor influencing the relationship between TyG-WC (*p* for interaction = 0.001), TyG-WHtR (*p* for interaction = 0.001), METS-IR (*p* for interaction = 0.005), and CVAI (*p* for interaction = 0.010) with SO. Similarly, gender was identified as a significant influencing factor influencing the relationship between CVAI and SO (*p* for interaction = 0.020).

### 3.5 Predictive performance of six surrogate IR indices for SO

**Figure 3:**
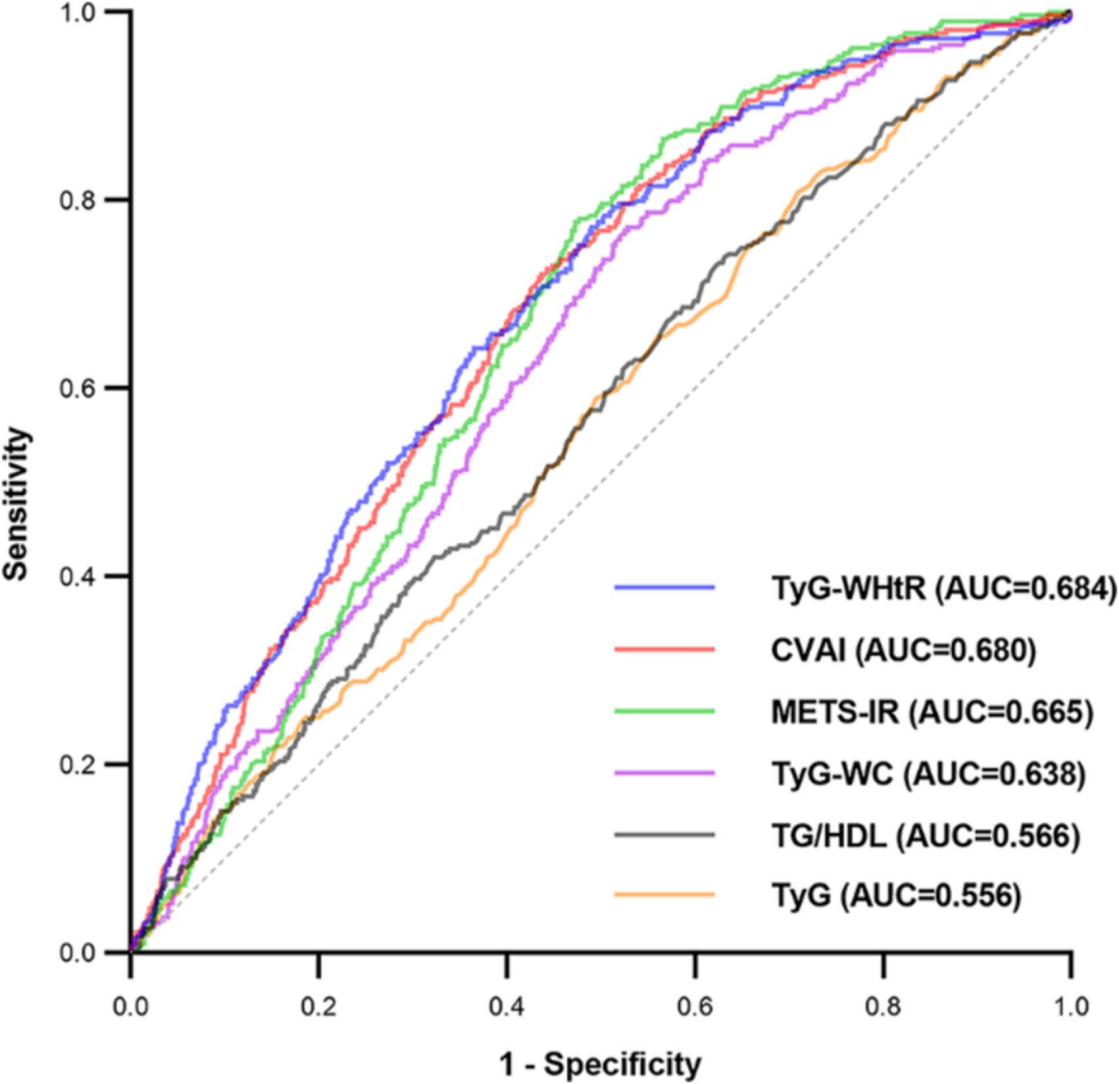
Receiver operating characteristic curves of surrogate IR indices for predicting SO. AUC, area under the curve; TyG, triglyceride-glucose; TyG-WC, triglyceride-glucose-waist circumference; TyG-WHtR, triglyceride-glucose-waist-to-height ratio; TG/HDL, triglyceride-to-high-density-lipoprotein-cholesterol ratio; METS-IR, metabolic score for insulin resistance; CVAI, Chinese visceral adiposity index.

The evaluation of the effectiveness of these IR surrogate indices in predicting SO is shown in Figure 3 and Supplementary Table S2. The TyG-WHtR index exhibited the highest prominent area under the curve (AUC) value of 0.684 (*p* < 0.001), surpassing the predictive capacities of other indices such as CVAI, METS-IR, TyG-WC, TG/HDL, and TyG. It was noteworthy that the optimal threshold value for TyG-WHtR for identifying SO in the middle-aged and older Chinese population was established at 458.778, with a sensitivity of 0.771 and a specificity of 0.510.

## 4 Discussion

In this nationwide cohort study, we identified significant positive correlations between the TyG, TyG-WC, TyG-WHtR, METS-IR, and CVAI indices, as well as the incidence of SO in middle-aged and older Chinese adults. The TyG index demonstrated a linear association with SO, whereas the TyG-WC, TyG-WHtR, METS-IR, and CVAI demonstrated nonlinear relationships. In terms of predictive SO, TyG-WHtR demonstrated optimal performance.

Surrogate indices of IR have been demonstrated to be effective tools for assessing IR levels in multiple studies (15, 29–33). However, research on surrogate IR indices with SO is scarce. Kim et al. conducted analyses on 3,821 older Korean people and found that, even after adjustment for multiple variables, a high TyG index was still associated with a higher risk of SO in both men and women (17). In contrast, in our study, the association between the TyG index and SO risk differed between men and women and was positively associated with SO in women (OR = 1.21, 95% CI: 1.02–1.43, *p* = 0.030) but not in men (OR = 1.15, 95% CI: 0.99–1.34, *p* = 0.076). The factors contributing to the gender-specific association between TyG and SO may include the following: First, the types of studies differed, as the research of Kim et al. was a cross-sectional study, whereas our study was a longitudinal cohort study. Second, the incidence of SO varies across diverse study populations. Since Kim et al. focused on older adults, a group with a high incidence of SO, their study was more likely to yield significant results. Our study, on the other hand, included a wide range of middle-aged and older people. Besides, the two studies adjusted for different covariates, which could also influence the study results.

Additionally, this study has broadened the investigation into the correlations between additional surrogate IR indices and SO risk. We created three models that adjusted for 20 potential confounding factors (age, gender, marital status, residence, education, smoking, alcohol consumption, sleep duration, comorbidities, and baseline CRP and creatinine). Our results indicated a significant positive connection between IR surrogates and SO risk. More importantly, the results of our study indicated that TyG-WHtR was the most suitable predictor for SO, and recent research has reported the critical role of TyG-WHtR in metabolic disorders and cardiovascular diseases. Xuan et al.’s population-based retrospective cohort study demonstrated that TyG-WHtR was deemed as the most optimal predictor for diabetes, compared to TyG and TyG-WC (34). Miao et al. demonstrated in a cross-sectional study involving 16,834 participants that TyG-WHtR outperformed TyG and other related parameters in identifying high cardiovascular risk (35). A systematic review and meta-analysis of 31 studies involving 123,231 individuals revealed that WHtR had the largest AUC for diabetes compared to WC and BMI (36). This could be attributed to the critical role of WHtR in metabolic disease.

Furthermore, this study demonstrated the nonlinear relationship between surrogate IR indices and SO risk and further refined and elucidated the specific trends of these indices in relation to SO risk through implementing two-piecewise logistic regression analysis. For TyG-WHtR values below 493.67, each increment in unit was associated with a rise of 1.7% in the likelihood of SO. However, once the value reached 493.67, the rate of risk increase slowed, with only a 0.6% increase in risk per unit increase. There was an approximately inverted U-shaped curve relationship between METS-IR and the risk of SO. In instances where METS-IR fell below 40.95, the likelihood of SO increased by 19.1% for every unit increase in METS-IR. In contrast, when METS-IR exceeded 40.95, there was no statistically significant change in the risk of SO for each unit increase in METS-IR. The TyG-WC, TG/HDL, and CVAI demonstrated saturation effects with SO risk by increasing it below specific inflection points. However, once they exceeded the inflection points, further increases did not result in an increase in SO risk. Consequently, the outcomes of this study provide a theoretical foundation for reducing surrogate IR indices in clinical practice, particularly when they are below the inflection points. Furthermore, the research provides specific recommendations for medical professionals to help patients with different surrogate IR index levels reduce their likelihood of developing SO.

Numerous studies have indicated that age and gender hold significant influence on the occurrence of SO (37). The subgroup analysis revealed that individuals aged 65 and above had a greater impact of TyG-WC, TyG-WHtR, METS-IR, and CVAI on SO risk. Previous studies have indicated that older individuals with diabetes tend to exhibit diminished muscle function and reduced muscle mass in comparison to their non-diabetic counterparts (38). However, the administration of insulin sensitizers has been shown to mitigate this condition (39). It is therefore possible that the increased correlation between IR and SO risk observed in the older population may be attributed to aging and its adverse effects on muscle and metabolic functions. Additionally, our study revealed a stronger correlation between CVAI and SO in females, possibly attributable to the influence of hormonal factors. A significant decline in estrogen levels associated with female menopause has been linked to impaired skeletal muscle function and lipid metabolism disorder, which ultimately leads to the development of SO (40, 41).

The primary strength of this study is the use of data from CHARLS, a nationally representative survey. This database is compliant with the highest international standards for data collection, which ensures comprehensive representativeness and high data quality. The findings regarding the association between IR surrogates and SO among the middle-aged and older population in China are both reliable and generalizable. Furthermore, this study not only comprehensively examined the correlation between IR surrogates and SO risk but also examined the nonlinear relationships using longitudinal data. Additionally, the study assessed the predictive capability of each indicator for SO risk, thereby deepening our understanding and application of IR surrogates in evaluating SO risk among middle-aged and older individuals. However, it is noteworthy that our study also possesses certain limitations. First, a validated formula specific to the Chinese population was employed to estimate the skeletal muscle mass in this investigation. Although this formula has previously demonstrated excellent consistency with DXA, it should not be considered a replacement for DXA or bioelectrical impedance for body composition measurement. Second, the follow-up period was restricted to 4 years due to the database containing only physical examination information from 2011 to 2015. To further reinforce the stability of the conclusions, extending the follow-up period is particularly necessary. Finally, although our study adjusted for numerous confounding factors, there remain unmeasured confounders, such as physical activity, nutritional status, and dietary intake, which may have affected the outcome.

## 5 Conclusion

This study provides compelling evidence that, among middle-aged and older Chinese individuals, the concentration of IR surrogates is positively correlated with the incidence of SO. It is notable that reducing these indicators, especially when they fall below the inflection points, might lower the risk of SO in clinical settings. In order to predict SO, TyG-WHtR might be the best indicator to use. Further study is necessary to confirm these findings spanning diverse ethnic communities and employing intervention strategies.

## Data Availability

The datasets analyzed for this study can be found in the China Health and Retirement Longitudinal Study, https://charls.pku.edu.cn/.

## Footnotes

^1^ https://charls.pku.edu.cn/
