# Supplementary material for: Longitudinal analysis of insulin resistance and sarcopenic obesity in Chinese middle-aged and older adults: evidence from CHARLS
Source: Front Public Health. 2024 Nov 18;12:1472456. doi: 10.3389/fpubh.2024.1472456 (PMC11609067; doi:10.3389/fpubh.2024.1472456)
Supplement: Supplementary file 1 [file Table_1.DOCX]

Supplementary Material

# Supplementary Figures and Tables

## Supplementary Figures

**Figure S1.** Flowchart of study participants selection.

Total participants at baseline of CHARLS in 2011 (n=**17708**)

Participants included in the final analysis (n=**6395**)

**11313** participants were excluded:

### No TyG, TyG-WC, TyG-WHtR, TG/HDL, METS-IR and CVAI data at baseline (n=**7958**)

### No information about age or age <45 (n=**339**)

### No SO data or diagnosed with SO at baseline (n=**508**)

### No information about SO in the follow-ups (n=**1988**)

### Abnormal values of BMI or waist (n=**178**)

### No information on demographics, health behaviors and chronic diseases (n=**342**)

With SO (n=**319**)

Without SO (n=**6076**)


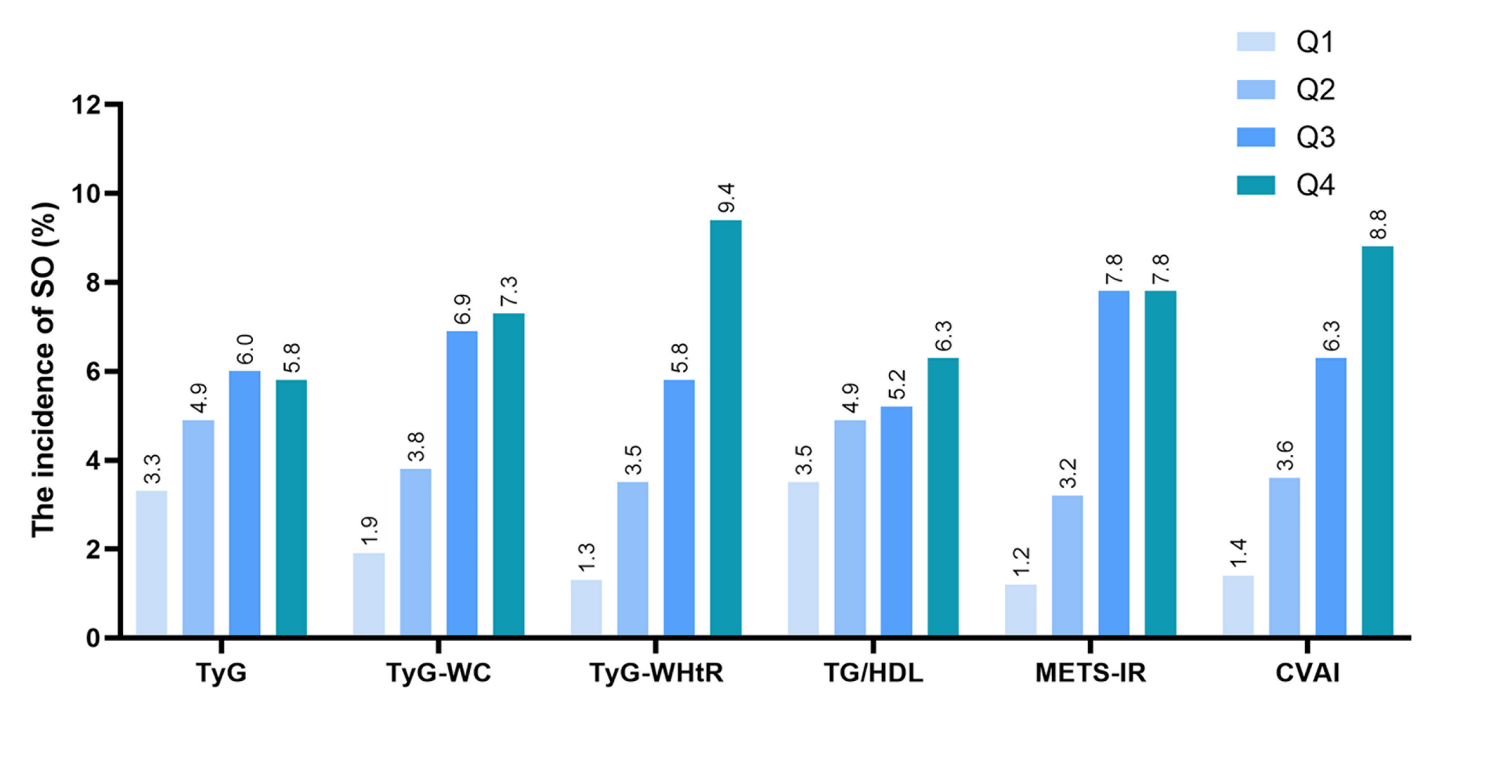


**Figure S2.** Incidence of SO across quartiles of six surrogate IR indices.

*SO* sarcopenic obesity, *TyG* triglyceride‐glucose, *TyG-WC* triglyceride‐glucose -waist circumference, *TyG-WHtR* triglyceride‐glucose -waist-to-height ratio, *TG/HDL* triglyceride-to-high-density-lipoprotein-cholesterol ratio, *METS-IR* metabolic score for insulin resistance, *CVAI* Chinese visceral adiposity index.

## Supplementary Tables

**Table S1.** Collinearity analysis.

| Variable | GVIF | DF | GVIF^(1/(2*Df)) |
| --- | --- | --- | --- |
| Age | 2.336 | 1 | 1.529 |
| Gender | 3.470 | 1 | 1.863 |
| Marriage | 1.110 | 1 | 1.054 |
| Residence | 1.096 | 1 | 1.047 |
| Education | 1.199 | 2 | 1.046 |
| Smoking | 1.907 | 1 | 1.381 |
| Drinking | 1.485 | 1 | 1.219 |
| Sleep duration | 1.084 | 1 | 1.041 |
| Hypertension | 1.225 | 1 | 1.107 |
| Diabetes Mellitus | 1.127 | 1 | 1.062 |
| Dyslipidemia | 1.202 | 1 | 1.096 |
| Cancer | 1.047 | 1 | 1.023 |
| Pulmonary disease | 1.038 | 1 | 1.019 |
| Heart disease | 1.123 | 1 | 1.059 |
| Stroke | 1.047 | 1 | 1.023 |
| Liver disease | 1.022 | 1 | 1.011 |
| Kidney disease | 1.046 | 1 | 1.023 |
| Depression | 1.164 | 1 | 1.079 |
| BMI | 21.189 | 1 | **4.603** |
| WC | 17.310 | 1 | **4.161** |
| CRP | 1.019 | 1 | 1.009 |
| HDL | 5.816 | 1 | **2.412** |
| LDL | 14.411 | 1 | **3.796** |
| TC | 17.142 | 1 | **4.140** |
| TG | 12.817 | 1 | **3.580** |
| Creatinine | 1.408 | 1 | 1.187 |

The collinearity analysis was conducted separately based on six surrogate IR indices (TyG, TyG-WC, TyG-WHtR, TG/HDL, METS-IR and CVAI), and the table presents the maximum GVIF among them. Collinearity analysis showed that BMI, WC, TC, TG, LDL, HDL, and six surrogate IR indices had high collinearity (GVIF^(1/(2*Df)) ≥ 2 indicates collinearity). *GVIF* generalized variance inflation factor, *DF* degree of freedom.

**Table S2.** ROC test of six surrogate IR indices for predicting SO.

|  | AUC | 95%CI | Cut off value | Specificity | Sensitivity | P Value | P value^#^ |
| --- | --- | --- | --- | --- | --- | --- | --- |
| TyG-WHtR | 0.684 | 0.657- 0.712 | 458.778 | 51.0 | 77.1 | **<**0.001 | Reference |
| TyG | 0.556 | 0.524- 0.587 | 8.276 | 29.1 | 80.9 | **<**0.001 | **<**0.001 |
| TG/HDL | 0.566 | 0.534- 0.597 | 3.203 | 38.8 | 76.9 | **<**0.001 | **<**0.001 |
| TyG-WC | 0.638 | 0.610- 0.665 | 714.420 | 47.9 | 76.5 | **<**0.001 | **<**0.001 |
| METS-IR | 0.665 | 0.640- 0.690 | 34.065 | 52.7 | 77.7 | **<**0.001 | 0.082 |
| CVAI | 0.680 | 0.653- 0.707 | 95.330 | 55.7 | 72.7 | **<**0.001 | 0.546 |

Note: P value^#^: comparison of TyG-WHtR and other five indices.

*AUC* area under the curve, *95%CI* 95% confidence interval, *TyG* triglyceride‐glucose, *TyG-WC* triglyceride‐glucose -waist circumference, *TyG-WHtR* triglyceride‐glucose -waist-to-height ratio, *TG/HDL* triglyceride-to-high-density-lipoprotein-cholesterol ratio, *METS-IR* metabolic score for insulin resistance, *CVAI* Chinese visceral adiposity index.
